# Multifunctional gold nanoparticles for osteoporosis: synthesis, mechanism and therapeutic applications

**DOI:** 10.1186/s12967-023-04594-6

**Published:** 2023-12-07

**Authors:** Weihang Gao, Chen Liang, Ke Zhao, Mingming Hou, Yinxian Wen

**Affiliations:** 1 Division of Joint Surgery and Sports Medicine, Department of Orthopedic Surgery, Zhongnan Hospital of Wuhan University, Wuhan, China; 2 Department of Orthopedics, Honghui Hospital, Xi’an Jiaotong University, Xi’an, China; 3 Department of Radiation and Medical Oncology, Zhongnan Hospital of Wuhan University, Wuhan, China; 4 Department of Orthopaedics, Wangjing Hospital, China Academy of Chinese Medical Sciences, Beijing, China

**Keywords:** Nanomaterial synthesis, Gold nanoparticles, Osteoporosis, Biocompatibility, Functionalization, Drug delivery, Osteoclast differentiation, Osteogenic differentiation

## Abstract

Osteoporosis is currently the most prevalent bone disorder worldwide and is characterized by low bone mineral density and an overall increased risk of fractures. To treat osteoporosis, a range of drugs targeting bone homeostasis have emerged in clinical practice, including anti-osteoclast agents such as bisphosphonates and denosumab, bone formation stimulating agents such as teriparatide, and selective oestrogen receptor modulators. However, traditional clinical medicine still faces challenges related to side effects and high costs of these types of treatments. Nanomaterials (particularly gold nanoparticles [AuNPs]), which have unique optical properties and excellent biocompatibility, have gained attention in the field of osteoporosis research. AuNPs have been found to promote osteoblast differentiation, inhibit osteoclast formation, and block the differentiation of adipose-derived stem cells, which thus is believed to be a novel and promising candidate for osteoporosis treatment. This review summarizes the advances and drawbacks of AuNPs in their synthesis and the mechanisms in bone formation and resorption in vitro and in vivo, with a focus on their size, shape, and chemical composition as relevant parameters for the treatment of osteoporosis. Additionally, several important and promising directions for future studies are also discussed, which is of great significance for prevention and treatment of osteoporosis.

## Background

Osteoporosis is a highly prevalent bone disease characterized by low bone mass and deterioration of bone tissue microarchitecture, which increases the risk of bone fragility and fractures; thus, it is a major concern for human health and public costs [1]. Globally, more than 200 million people are affected by osteoporosis, and it is predicted that osteoporotic fractures will encompass fifty percent of people with fractures by 2050 [2]. In recent years, various therapeutic drugs, such as oestrogen, bisphosphonates, teriparatide, and RANK ligand inhibitors, have been introduced to the market for the treatment of osteoporosis. However, their clinical applications are limited due to the adverse effects and the high costs associated with these medications. Among the available treatment options, oral bisphosphonates (BPs) are widely used for osteoporosis management. Multiple adverse effects can occur and contribute to low adherence to BPs therapy, which sometimes requires evaluation by a gastroenterologist. Additionally, most drugs are systemically administered, and their low bone-targeting efficiency often necessitates high doses and frequent administration, which can also lead to serious adverse effects [3–5]. Given these challenges, there has been growing interest in developing new approaches for the prevention and treatment of osteoporosis. Researchers are exploring alternative therapies that can improve treatment efficacy while minimizing adverse effects and reducing treatment costs.

Nanomaterials have been extensively studied for various biomedical applications, including diagnosis, therapy, drug delivery, and imaging of different diseases (both in vitro and in vivo) [6–10]. This is primarily due to their unique physicochemical properties, such as small size, large surface area, relatively high surface area-to-volume ratio, high stability, and unique optical properties. Among the different nanomaterials that are used in biomedicine, gold nanoparticles (AuNPs) are particularly notable [11–14]. AuNPs are colloidal particles composed of gold atoms with diameters ranging from several nanometres to several hundred nanometres and are widely used in materials science, industrial catalysis, bioanalytical chemistry, biomedicine, and other fields [11, 15]. AuNPs possess several advantageous characteristics that make them suitable for biomedical applications [16]. For example, they are chemically inert, thus allowing for easy functionalization and modification, and they can be readily synthesized in various sizes and shapes, thus offering versatility for different applications. Via classical methods such as the Frens and Brust methods [17], highly monodisperse AuNPs with diameters ranging from 1 to 150 nm can be prepared on a large scale. Furthermore, the particle size and shape of AuNPs can be finely controlled, thus enabling for customization for specific applications. The small particle size of AuNPs enables their wide distribution in vivo, which allows them to interact with biological systems while also exhibiting excellent biocompatibility and reducing the likelihood of adverse reactions. Furthermore, the large specific surface area of AuNPs provides ample opportunities for functionalization and modification, thus allowing the attachment of various chemical groups, such as sulfhydryl, phosphorylated hydrogen, and amine modifications [18]. This scenario enables the development of AuNPs with targeted functions, whereby the attached ligands can specifically recognize and bind to particular molecules or cells of interest. Moreover, by attaching therapeutic agents to their surfaces or by encapsulating drugs within their structures, AuNPs can act as carriers, thus facilitating targeted and controlled drug release at specific sites in the body [19, 20]. By leveraging unique physicochemical properties and drug delivery mechanisms, AuNPs can be utilized in various therapeutic approaches, such as photothermal therapy, photodynamic therapy, immunotherapy, targeted therapy, gene therapy, and combination therapies [21–24].

Remarkably, many studies have demonstrated that AuNPs can regulate osteogenesis and osteoclastic activity, as well as attenuate adipogenic differentiation, as evidenced by in vitro and in vivo studies [25–27]. Therefore, AuNPs could be promising biomaterials for bone homeostasis regulation in osteoporosis. Several factors, such as size, shape, surface topography, surface chemistry, and physical characteristics, have been found to significantly influence the capability of AuNPs to regulate osteogenesis, which provides potential strategies for the upgrade and revamping of AuNP products [28–30]. Subsequently, we summarize the studies on the mechanisms underlying the regulation of bone homeostasis by AuNPs. Previous studies have shown that AuNPs exhibit potential in inhibiting excessive bone resorption and in promoting the repair of bone defects by regulating the acid secretion function of osteoclasts [31]. Additionally, AuNPs have been shown to promote the osteogenic differentiation of bone marrow mesenchymal stem cells into osteoblasts, thus enhancing cellular osteogenesis [32, 33].

Despite the unique advantages of AuNPs, they are not without limitations. Gold particles in the nanometric range can manifest toxicity, which is contingent on factors like their size, shape, surface charge, and functionalization [34]. Most AuNPs with sizes ranging from 5 to 100 nm are challenging to eliminate from the body due to their large size. These larger AuNPs are prone to be trapped by the reticuloendothelial system, leading to low uptake in bone tissue and inevitable accumulation in organs such as the liver and spleen [35, 36]. Reducing the particle size could facilitate the evasion of reticuloendothelial absorption. Recent studies have demonstrated that AuNPs with sizes below 20 nm can effectively escape reticuloendothelial absorption and exhibit good cellular uptake [37]. However, these particle sizes still exceed the renal clearance barrier of 5.5 nm, which raises concerns about potential long-term accumulation and toxicity in the kidneys or other parts of the renal system [38, 39]. Additionally, AuNPs do not possess inherent targeting abilities, which limits their effectiveness in targeted drug delivery. Excessively high concentrations of AuNPs can lead to cell death, while too low concentrations may have minimal influence on differentiation. To enhance the absorption and distribution in bone tissue and reduce the required dosage of gold particles, surface chemical modification of gold particles can improve their targeting ability [40, 41]. Therefore, the pursuit of gold particles with suitable sizes, shape and optimal targeting ability has become a critical consideration in the development of AuNPs-based pharmacological treatments.

Overall, the exceptional properties of AuNPs make them promising candidates for a wide range of biomedical applications, thus providing new possibilities for improved diagnostics, therapies, and personalized medicine. Herein, we offer insights into the most recent and promising research developments concerning AuNPs prepared for osteoporotic and further biomedical applications.

## Synthesis of gold nanoparticles

In 1951, the Turkevich method was first reported for the synthesis of AuNPs [42]. This method is widely used for producing spherical AuNPs and typically yields particles with sizes ranging from 1 to 2 nm. The principle behind this technique involves the reduction of gold ions (Au3 +) to gold atoms (Au0) using various reducing agents, such as citrate, amino acids, ascorbic acid, or UV light [43–45]. Another method was first developed in 1994 and is known as the Brust method [17]. This method enables the synthesis of AuNPs with sizes ranging from 1.5 to 5.2 nm through a two-phase reaction. The Brust method involves the use of phase transfer agents (such as tetraoctylammonium bromide) to transfer the gold salt from an aqueous solution to an organic solvent. Afterwards, the gold salt is reduced by using reducing agents such as sodium borohydride, along with the addition of an alkanethiol. The alkanethiol serves the purpose of stabilizing the synthesized AuNPs. As a result of this reaction, the colour of the solution changes from orange to brown, thus indicating the successful formation of AuNPs.

The aforementioned Turkevich and Brust methods are primarily used for synthesizing spherical AuNPs. However, for the production of AuNPs with various geometries and shapes, such as nanorods, nanocages, and nanostars, seed-mediated growth methods are employed [32, 46–48]. Gold nanocages are hollow gold nanoparticles with a cage-like structure. Moreover, they have a high specific surface area, excellent surface modifiability, and a high drug-loading rate, thus making them ideal for drug delivery [49, 50]. The use of different concentrations of gold seed solution resulted in gold nanostars and nanorods with different diameters. The synthesized gold nanostars and nanorods exhibited different biological activities and physicochemical properties, thus rendering them advantageous for diverse applications [30, 51]. Seed-mediated growth stands as a dependable technique for fabricating differently shaped AuNPs, yet multiple variables influence the dimensions of these AuNPs and hence require meticulous and tedious regulation. An investigation demonstrated that employing elevated concentrations of HAuCl4 or elevated temperatures resulted in larger gold nanorods with lower aspect ratios [52]. Various parameters can influence the size of nanomaterials, which thus should be carefully controlled during the synthesis progress. The Brust method offers a straightforward approach to creating stabilized AuNPs with well-controlled sizes and minimal variability. However, a potential limitation of this method lies in the production of less-dispersed AuNPs and the use of organic solvents, thus constraining their applicability in biological contexts [53]. Overall, chemical methods that are used for the synthesis of AuNPs are associated with certain limitations, including concerns related to environmental impacts and biocompatibility [54]. Some of the chemicals employed in the synthesis process may have adverse effects on the environment and can pose health risks when administered to living organisms, thereby restricting the biological applications of such nanoparticles. To address these concerns, various biological methods have been developed for the synthesis of AuNPs. Microorganisms and plants have been identified as being suitable materials for the biogenic synthesis of nanomaterials [55–58]. The extracellular synthesis of AuNPs involves the trapping of gold ions on the surface of cells, and it is facilitated by membrane enzymes that are responsible for their attachment to the cell membrane. Phyto-nanotechnology has also gained significant interest as an environmentally friendly, cost-effective, and rapid approach for synthesizing gold nanoparticles using plant extracts. Numerous studies have reported of the use of different plants or plant extracts to biologically synthesize AuNPs. This approach utilizes nonhazardous biological components from plants to reduce and coat AuNPs, thereby reducing waste generation and minimizing the need for additional purification steps [59]. Although various plant parts have been explored for the successful synthesis of AuNPs, leaves are the most commonly used plant component [60].

Chemical synthesis methods, such as the reduction of gold ions using citrate or other reducing agents, offer convenience and produce size controllable nanoparticles. Although the chemical synthesis route is an easier method, the associated potential risks of secondary reaction products pose severe concerns to both the environment and human health. In contrast, biological sources provide molecules that play an important role in the reduction and stabilization of AuNPs. Biologically reduced AuNPs offer enhanced stability and safety compared to chemically reduced counterparts, thus making them a favourable and effective method in various applications. However, biological synthesis methods also possess their own limitations, which include difficulty in screening for reactive components and nonhomologous pathogenic concerns [61, 62]. Moreover, cells and microbes take considerable time to grow; thus, the overall synthesis process can become complicated and time-consuming. To address these limitations, a potential solution could entail amalgamating the advantages of both chemical and biological approaches. Researchers could explore hybrid synthesis techniques that leverage the simplicity and reproducibility of chemical methods while incorporating environmentally friendly and biocompatible components derived from biological sources [63–65]. By optimizing the reaction conditions and precisely controlling the reaction kinetics, it may be possible to achieve more uniform and controllable AuNPs with reduced waste generation and enhanced biocompatibility. From this, integrating green chemistry principles into the synthesis process could reduce the use of hazardous reagents and minimize the overall environmental impact. Therefore, the choice of the most suitable synthesis procedure for future AuNP applications will depend on the specific research questions and considerations.

## Relationship between the size and shape of AuNPs and osteoporosis

**Fig. 1:**
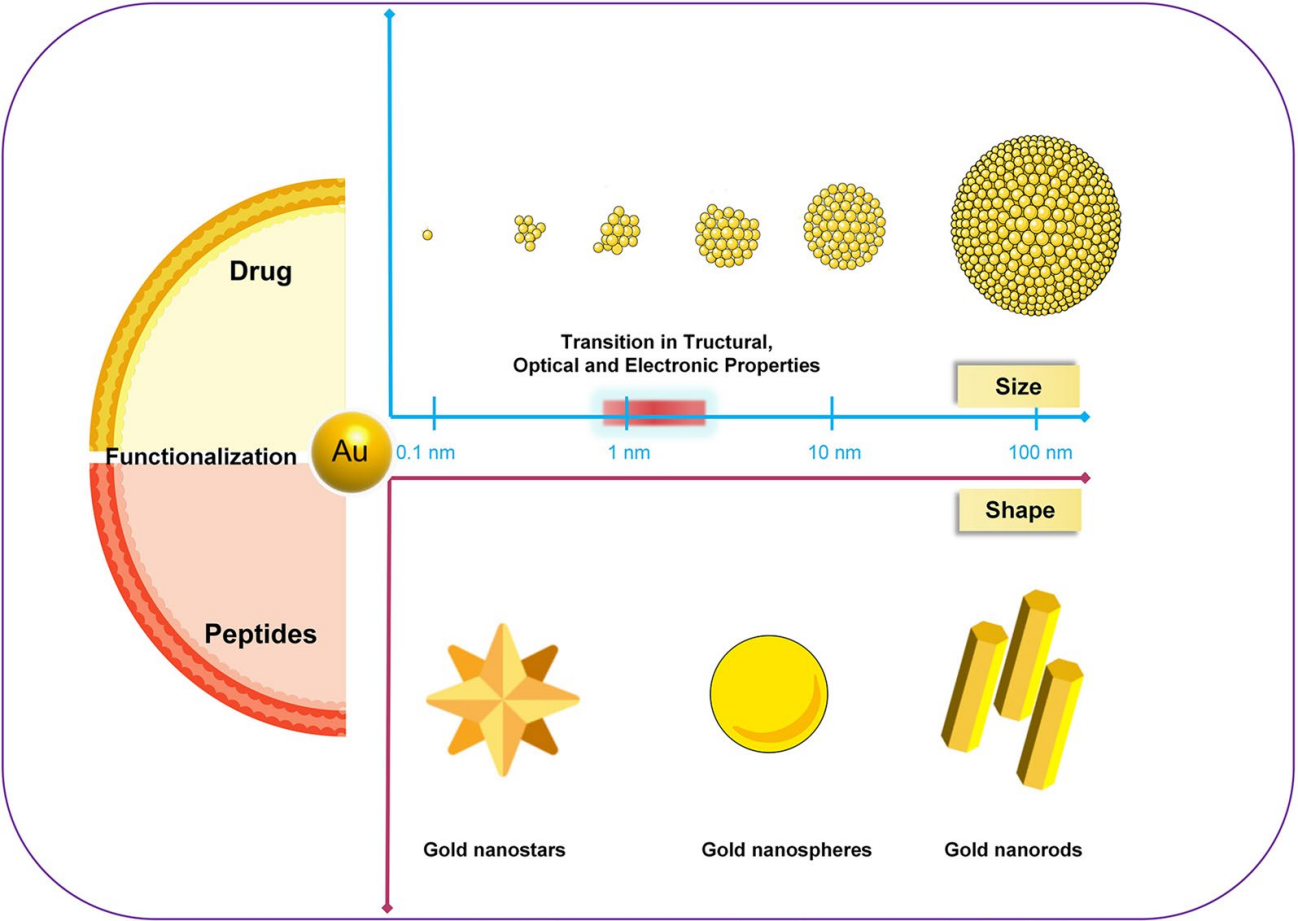
Different sizes, shapes, and ligands of AuNPs

In 1857, Michael Faraday discovered the light-scattering properties of suspended gold microparticles, a phenomenon now referred to as the Faraday-Tyndall effect [66]. Years later, Hirsh et al. found that AuNPs irradiated with an electromagnetic wavelength of 820 nm could generate local hyperthermia, which showed potential for solid tumor treatment [67]. The size of AuNPs determines the distribution of surface charges and the polarization of free electrons. Various morphologies of AuNPs have been synthesized, thus allowing for the absorption/scattering peak to be shifted to the near-infrared window, which enables AuNPs to receive light energy in deep tissues. Over the past two decades, numerous studies have focused on different shapes of AuNPs, including nanospheres, nanorods, nanostars, nanoshells, nanocages, and nanoplates [32, 68–70]. These AuNPs have been extensively investigated for disease diagnosis and treatment. In July 2019, the U.S. Food and Drug Administration (FDA) made a significant milestone by approving an oral drug based on AuNPs for the treatment of amyotrophic lateral sclerosis [71], thus further demonstrating the safe and promising use of AuNPs in disease treatment. In the context of osteoporosis treatment, gold nanoparticles have shown effectiveness without causing toxic effects. Moreover, they have been found to promote osteoblast differentiation [72], inhibit osteoclast formation [73], and block the differentiation of adipose-derived stem cells [33]. Specifically, gold nanospheres, nanostars, and nanorods have been widely utilized in the treatment of osteoporosis (Fig. 1) [74–76]. Also, various ligands, functional groups, and key features with AuNPs (as shown in Table 1) are used to achieve abundant and stronger functions in bone tissue engineering.

**Table 1:** Characteristics of conjugated AuNPs regulating osteoclast/osteogenic processes

Particle name	Size	Shape	Ligands/methods	Model/cell type	Properties	References
Gold nanoparticles	150 nm	Sphere	Electrical explosion	BMMs	Inhibits osteoclast formation	[103]
GNS	60 nm	Sphere	Citrate	Mice	Inhibits osteoclast formation	[27]
CUR-CGNPs	20 to 40 nm	Sphere	β-cyclodextrin	BMMs	Inhibits osteoclast formation	[101]
AuNPs	13.5 ± 0.3 nm	Sphere	Citrate	BMMs	Inhibits osteoclast formation	[73]
BSA-Au clusters	1.63 ± 0.31 nm	Sphere	BSA	BMMs	Inhibits osteoclast formation	[87]
GNPs	15,30,50,75,100 nm	Sphere	Citrate	hADSCs	Promoting osteogenic differentiation	[78]
AuNPs	40,70,110 nm	Sphere, Nanostars, Nanorods	Citrate	hMSCs	Promoting osteogenic differentiation	[32]
GNSs	40.3 ± 2.0 nm	Nanostars	Seed-mediated	hMSCs	Promoting osteogenic differentiation	[89]
GNRs	NA	Nanorods	CTAB	BMSCs	Promoting osteogenic differentiation	[90]
AuNPs	5, 13, 45 nm	Sphere	Citrate	hPDLPs	Promoting osteogenic differentiation	[79]
AuNPs	40 nm	Sphere	Chitosan	hADMSCs	Promoting osteogenic differentiation	[120]
AuNPs	20 ± 2 nm	Sphere	Citrate	MSCs	Promoting osteogenic differentiation	[100]
AuNPs	13.22 nm	Sphere	NA	hPDLSCs	Promoting osteogenic differentiation	[72]
AuNPs	4, 40 nm	Sphere	Citrate	hMSCs	Promoting osteogenic differentiation	[29]
Lys-AuNCs	1.0 to 3.0 nm	Sphere	Lysozyme	MC3T3-E1	Promoting osteogenic differentiation	[86]

hPDLSCs: human periodontal ligament stem cells; hADMSCs: human mesenchymal stem cells; BMMs: bone marrow-derived macrophages; MSCs: mesenchymal stem cells; ADSCs: human adipose-derived stemcells; hMSCs: human bone marrow-derived mesenchymal stem cells; hPDLPs: human periodontal ligament progenitor cells; CTAB: cetyl trimethylammonium bromide; NA: respective information not provided in the articles

### AuNPs of different sizes

We utilize gold nanoparticles for their ease of administration via intravenous injection due to their small size and high aqueous solubility. One critical factor influencing the clinical utility of nanoparticles is their cellular uptake capability. The size of most gold nanomaterials is within 100 nm, which allows us to further investigate the relationship between their size and potential pharmacodynamic effects. Recent studies have provided information on the preferential uptake of different sizes of gold nanoparticles by pancreatic cancer cells [77]. Specifically, 20 nm gold nanoparticles exhibited higher cellular uptake than 10 nm, 30 nm, 40 nm, 50 nm, and 100 nm gold nanoparticles. This higher uptake rate correlated with a stronger pharmacodynamic effect on the cells, thus indicating that both excessively large and excessively small gold nanoparticles have a negative impact on cellular uptake. In the field of osteogenesis research, various sizes of gold nanoparticles, including 15 nm, 30 nm, 50 nm, 70 nm, and 100 nm sizes, have been applied. The results demonstrated that 30 nm and 50 nm gold nanoparticles exhibited not only the highest cellular uptake rate but also the most pronounced osteogenic effect in osteogenesis assays, as indicated by alkaline phosphatase activity and alizarin red staining [78].

Several studies have demonstrated contrasting effects of gold nanoparticles with different sizes on cell growth and osteogenesis. For instance, 4 nm gold clusters have been shown to exert a significant inhibitory effect on cell growth compared to 40 nm gold nanoparticles. Further analysis demonstrated that 40 nm nanoparticles exhibited a positive osteogenic effect, and 4 nm gold nanoclusters exhibited an inhibitory effect on osteogenesis due to their stimulation of high levels of cellular reactive oxygen species (ROS) [29]. In a cross-sectional comparison involving AuNPs with different diameters (5 nm, 13 nm, and 45 nm) for promoting osteogenesis, it was concluded that nanoparticles larger than 10 nm, such as 13 nm and 45 nm, could enhance cellular osteogenesis [79]. In particular, it was discovered that 45 nm AuNPs significantly improved the osteogenic activity and inhibited the osteoclastogenesis activity of human periodontal ligament cells (hPDLCs). As a result, there was more newly formed periodontal attachment, which increased bone and cementum and reduced tissue destruction during the progression of periodontitis. This effect was supported by a significant increase in osteogenesis-related proteins, including Runx2, ALP, COL1, and OPN. Spherical 45 nm AuNPs were synthesized by using chemical methods in a similar study [28], in which L-cysteine or D-cysteine was attached to the AuNPs to investigate their potential for osteogenic differentiation. Consistent with previous findings, the AuNPs exhibited a significant ability to induce osteogenic differentiation in hPDLCs, as evidenced by the increased levels of both mRNA and proteins of osteogenesis-related genes. Moreover, L-Cys-AuNPs demonstrated superior performance compared to D-Cys-AuNPs in the osteogenic activity of hPDLCs.

AuNPs that are too large in size (> 50 nm) result in reticuloendothelial capture, which leads to reduced uptake by bone tissue and higher doses of the required gold nanoparticles [35, 36, 80]. Trapped gold nanoparticles will accumulate in the hepatic and renal tissues [81]. However, excessively high concentrations of gold nanoparticles induce cytotoxicity, thus leading to cell necrosis and degeneration. To overcome these limitations, researchers have explored the use of smaller gold nanoparticles to evade reticuloendothelial capture and improve their overall utilization. Gold nanoparticles with sizes below 10 nm are referred to as small gold nanomaterials. They exhibit distinct physical and chemical properties compared to larger gold nanomaterials. Furthermore, gold nanomaterials smaller than 3 nm belong to the "Ångstrom dimension" category and exhibit a very light or nearly transparent colour in solution. Notably, under ultraviolet light irradiation, they can display characteristic fluorescence wavelengths [82]. Gold nanoclusters (AuNCs) are Ångstrom-scale gold nanoparticles characterized by their ultrasmall size (typically ≤ 3 nm) [83]. The structure of AuNCs consists of a metallic core comprising a few gold atoms surrounded and protected by Au coordinating ligands. Consequently, their electronic, magnetic, and optical properties differ from those of large-sized AuNPs [84]. The absorbance properties of gold particles in solution are highly influenced by their nanoparticle diameter. Ultrasmall AuNPs or nanoclusters with discrete sizes, such as Au102 and Au144 (less than 3 nm in diameter), do not exhibit the characteristic strong surface plasmon resonance (SPR) absorption peak that is typically observed at approximately 500–550 nm [85]. Ultrasmall gold nanomaterials composed of several to a few hundred gold atoms exhibit extremely low cytotoxicity and exceptional red fluorescence characteristics. These unique characteristics allow them to effectively circumvent the autofluorescence background in vivo, making them more advantageous compared to larger gold nanoparticles (Fig. 1).

In Zhang’s study, small AuNPs were synthesized at a concentration of 0.25 mM by using citrate as a reducing agent, after which the AuNPs were capped with L-cysteine. In this study, it was found that 5 nm gold nanoparticles exhibited a significant inhibitory effect on osteogenesis rather than an osteogenic effect on human periodontal ligament stem cells [79]. In a similar study, spherical methoxy-poly (ethylene glycol)-capped AuNPs (Au-mPEG NPs) with an average diameter of 4.0 nm were synthesized using trisodium citrate as the reducing agent. Interestingly, these Au-mPEG NPs were found to downregulate osteogenic gene expression and upregulate adipogenic gene expression in human mesenchymal stem cells (hMSCs), suggesting their potential role in modulating cell differentiation pathways [29]. These findings suggest that small gold nanoparticles may have a negative impact on bone tissue by inhibiting osteogenic differentiation and promoting lipogenic differentiation. However, it is important to note that there are different results reported in other studies. For instance, ultrasmall gold nanoclusters (Lys-AuNCs) (with an average diameter of 2.0 nm) were synthesized by using lysozyme as a protective template and were found to stimulate osteogenic differentiation [86]. In this particular study, Lys-AuNCs were shown to promote osteogenic differentiation and reduce osteoclast activity. Interestingly, the lysozyme itself, individual AuNPs, or a mixture of lysozyme and AuNPs had minimal effects on osteoblastic differentiation compared to the Lys-AuNCs. In another study, it was observed that the hydrodynamic size of the synthesized BSA-Au cluster was approximately 2.2 nm, which was slightly larger than that of the BSA protein [87]. Studies conducted in MDA-MB-231 cells demonstrated that BSA-Au clusters suppressed the expression of osteolysis-related factors. Furthermore, the BSA-Au clusters inhibited the subsequent activation of the NF-κB pathway in BMMs (bone marrow-derived macrophages). These findings suggest that BSA-Au clusters have the potential to mitigate osteolysis and modulate the NF-κB pathway, thus highlighting their possible therapeutic applications in the context of bone-related diseases.

These contrasting findings highlight the complex nature of the interactions between gold nanoparticles of different sizes and bone tissue, and further research is needed to elucidate the underlying mechanisms. It is worth noting that the different ligands used for surface functionalization may also result in the differential osteogenic effects that are observed among gold nanoparticles. Small gold nanoparticles functionalized with various surface ligands have also demonstrated excellent osteogenic differentiation ability. Therefore, these novel functionalized gold nanoparticles hold promise as potential therapeutics for osteoporosis.

### AuNPs in different shapes

#### Gold nanospheres

Gold nanospheres are spherical gold colloidal particles formed by the aggregation of a large number of gold atoms, which is also the most common morphology of gold nanoparticles. The data have consistently shown that gold nanospheres of various sizes, including 15 nm, 30 nm, 50 nm, 75 nm, and 100 nm, effectively promoted the differentiation of adipose-derived stem cells (ADSCs) into osteoblasts compared to the control group. However, the size of gold nanospheres was found to have varying effects on the osteogenic differentiation of ADSCs. Among gold nanospheres ranging from 15 to 100 nm, the 30 nm and 50 nm gold nanospheres exhibited significantly better osteogenic effects than other particle sizes. On the other hand, a recent study has shown that gold nanospheres with a diameter of 50 nm demonstrate maximum cellular uptake, and the uptake is significantly higher than that of gold nanorods when the sizes are comparable [78, 88]. Conversely, 100 nm gold nanospheres exhibited the lowest osteogenic differentiation effect. In another study, it was observed that the ALP concentration in groups treated with 40, 70, and 110 nm gold nanospheres, as well as 70 nm gold nanorods, was significantly higher than that of the control groups. Conversely, treatment with 40 nm gold nanorods resulted in a noticeable reduction in ALP concentration compared to the control. Gold nanoparticles of varying sizes and shapes may influence the osteogenic differentiation of hMSCs by modulating nuclear YAP activity [32, 89]. The findings indicated that 40 nm gold nanospheres and 70 nm gold nanorods promoted nuclear YAP activity, whereas 40 nm gold nanorods inhibited this activity. The groups treated with 40 nm gold nanospheres exhibited higher percentages of hMSCs with nuclear localized YAP, which may enhance the early osteogenic differentiation of hMSCs by upregulating the expression of certain early osteogenic marker genes, including RUNX-2, OST, and OCN. The mechanism investigation suggests that the size and shape of AuNPs may modulate the osteogenic differentiation of hMSCs by regulating nuclear YAP activity.

#### Gold nanostars

Gold nanostars are a unique type of nanoparticle distinguished by their multibranched surface structures. The optical properties of gold nanostars can be tuned by adjusting their size and degree of branching, thus allowing them to exhibit enhanced optical responsiveness in the near-infrared range, which is advantageous for various medical diagnostic and therapeutic applications. Moreover, the cytotoxicity of gold nanostars is influenced by factors such as size, shape, and chemical modifications. Notably, a recent study suggested that gold nanostars exhibit lower cytotoxicity than gold nanospheres of similar size [30]. In another study that investigated the cellular uptake capacity and osteogenic differentiation potential of gold nanostars, it was observed that the cellular uptake capacity of gold nanostars was significantly increased with increasing particle size ranging from 40–110 nm [32]. However, contrasting results were obtained in osteogenic differentiation experiments, wherein gold nanostars exhibited weaker osteogenic ability than gold nanospheres and gold nanorods. In another study, the effects of dendritic polyglycerol-conjugated gold nanostars (dPG@GNS) with different densities of ligands on the osteogenesis of MSCs were systematically investigated [89]. The data demonstrated that all dPG@GNS promoted osteogenic differentiation of MSCs, as evidenced by increased alkaline phosphatase activity, calcium deposition, and expression of osteogenic proteins and genes, including ALP, Runx2, and OCN. Moreover, the various densities of functional groups on dPG@GNS nanocomposites facilitated the nuclear localization of Yes-associated protein through mechanical stimuli, thus leading to the upregulation of osteogenic differentiation in MSCs. These findings highlight the potential of gold nanostars nanocomposites in promoting osteogenesis.

#### Gold nanorods

Gold nanorods exhibit unique characteristics with their short- and long-axis orientations and dual wavelengths [48]. Several studies have been conducted to investigate the cellular uptake of AuNPs by varying their size, shape, and surface chemistry. For instance, gold nanospheres with a diameter of 70 nm have been found to exhibit the highest cellular uptake, surpassing that of gold nanorods of similar size [32, 78]. Another study supported this finding, thus indicating that the cellular uptake of Au nanorods is highly influenced by their aspect ratio because higher aspect ratios often lead to lower uptake [88]. In addition, another study investigated the cellular uptake capacity and osteogenic differentiation capacity of gold nanorods sized from 40 to 110 nm. It was observed that the cellular uptake capacity of gold nanorods significantly increased with particle size, and the uptake capacity of larger gold nanorods was notably higher than that of gold nanospheres of similar sizes [32]. However, different results were observed in the osteogenic differentiation experiments, with 70 nm gold nanostars showing the highest osteogenic differentiation ability in the alizarin red and ALP staining experiments. Conversely, rod-shaped gold nanoparticles with a size of 40 nm displayed the lowest osteogenic effect and exhibited inhibitory effects on osteogenesis compared to the blank control. In one specific study, the seed-induced growth method was employed to synthesize gold nanorods, which required a substantial amount of cetyl trimethylammonium bromide (CTAB) as ligands to stabilize their shape [90]. To enhance the biocompatibility of gold nanorods and to reduce the immune response, they were further modified with endogenous proteins. The modified gold nanorods (known as eP@GNRs) exhibited effective promotion of osteogenic differentiation in bone marrow mesenchymal stem cells (BMSCs) when exposed to 808 nm laser irradiation. This effect was attributed to the photothermal properties of eP@GNRs. Additionally, the study provided information on the key osteogenic pathways activated by eP@GNRs in BMSCs, which included the MAPK, Akt, Smad, and Wnt/β-catenin pathways, with all of these pathways playing significant roles in regulating osteogenic differentiation processes.

## Effect of AuNPs on bone homeostasis

Bone, which represents the central axis system of the body, serves multiple functions, including providing physical support and protection, participating in calcium metabolism and endocrine regulation, and facilitating the hematopoietic system in the bone marrow [91]. Throughout adulthood, bone undergoes continuous remodelling in response to normal wear and tear, mechanical forces, and the aging process. During this remodelling process, the damaged or failing microstructure of the bone is removed by cells such as osteoclasts and replaced with new bone tissue by cells such as osteoblasts [92]. Bone remodelling is an ongoing dynamic process that involves both bone formation and bone resorption activities, which are typically balanced to maintain bone homeostasis [93]. The equilibrium between osteoblasts and osteoclasts is crucial for maintaining bone health and stability [94]. Moreover, the disruption of this balance leads to various bone metabolic diseases [95]. Excessive activity of osteoclasts can contribute to the development of conditions such as osteoporosis, Paget's disease, and rheumatoid arthritis. Osteoporosis is characterized by a reduction in bone density and strength, thus making bones more prone to fractures. Paget's disease involves the abnormal breakdown and formation of bone tissue, thus leading to weakened and deformed bones. Additionally, rheumatoid arthritis is an autoimmune disease that causes inflammation in the joints, which can lead to bone erosion over time. In summary, bone remodelling is a continuous process in which damaged bone is removed and replaced with new bone tissue. The balance between osteoblasts and osteoclasts is essential for maintaining bone homeostasis, and disruptions in this balance can contribute to various bone metabolic diseases.

**Fig. 2:**
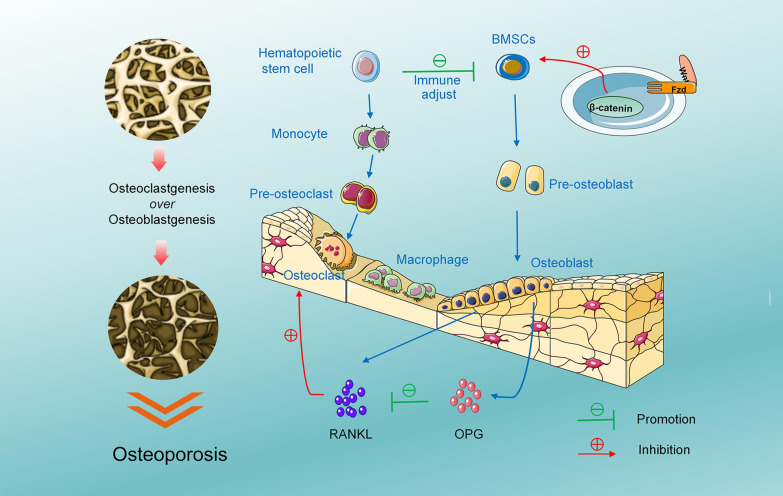
Schematic representation of bone remodelling and underlying pathogenetic mechanisms. During bone remodelling, osteoclasts are derived from hematopoietic cells, while osteoblasts are derived from bone marrow mesenchymal stem cells (BMSCs). The Wnt/β-catenin signalling pathway activates its ligands, thus promoting bone formation. The regulation of osteoclastogenesis involves the interaction between osteoblasts and osteoclast precursors. Osteoclast differentiation is promoted when the receptor activator of RANK (expressed by osteoclast precursors) binds to its ligand RANKL. However, osteoprotegerin (OPG), which is secreted by osteoblasts, competes with RANK for binding to RANKL, thereby reducing signalling to osteoclasts. This negative feedback loop suppresses osteoclast differentiation, thus making the OPG/RANK/RANKL system crucial for regulating osteoclast activity and differentiation

Osteoclast differentiation begins with the activation of BMMs stimulated by receptor activators of nuclear factor-κB ligand (RANKL) and macrophage colony-stimulating factor (M-CSF) [96]. Osteoblasts release both RANKL and osteoprotegerin (OPG) to regulate bone homeostasis (Fig. 2). RANK, which is the receptor for RANKL, is expressed on osteoclasts. When RANK interacts with RANKL, it initiates downstream signalling pathways such as NF-κB, MAPK, and AKT, thus leading to the expression of genes associated with osteoclast formation [97]. Furthermore, osteoclastogenesis generates ROS, which can activate signalling pathways such as NF-κB and MAPK, thus contributing to osteoclast differentiation and bone resorption [98]. Moreover, OPG competes with RANK to bind with RANKL, thus reducing RANKL signalling and maintaining a balance in bone resorption [99]. Variations in the OPG gene have been shown to be closely linked to the pathogenesis of osteoporosis. The interaction between RANKL and its receptor RANK triggers downstream signalling pathways that activate the expression of genes necessary for osteoclast formation. The balance between RANKL and OPG is crucial for maintaining bone homeostasis by regulating the activity of osteoclasts and osteoblasts.

AuNPs have demonstrated the ability to modulate the differentiation of several cell lines by influencing both osteogenic and osteoclastic differentiation processes. An understanding of the underlying mechanisms of AuNP interactions with cells is crucial for developing effective strategies to address osteoporosis. Previous studies have explored the interaction between AuNPs and cells, and they have provided insight into the fundamental mechanisms of cell differentiation and self-renewal in their presence. Herein, we summarize the mechanisms of cellular osteogenic and osteoclastic differentiation regulation induced by AuNPs. Upon incubation with cells, the AuNPs interact with the membrane and are internalized through endocytosis or other pathways. Once inside the cytoplasm, AuNPs interact with bioactive substances, such as proteins, thus initiating a range of cellular metabolic processes [78, 100]. Importantly, the mechanical stress exerted on cells by AuNPs has been found to play a vital role in stimulating cellular metabolism [101]. Several signalling pathways, including NF-κB, MAPK, and Wnt/β-catenin, have been reported to be involved in the interaction between AuNPs and different cell lineages, which will be discussed in this review.

### Role of AuNPs in bone resorption

The development of osteoporosis is closely associated with osteoclasts (Fig. 2), as their increased activation and differentiation result in enhanced bone resorption, thus leading to systemic bone loss [96]. Osteoclasts are specialized cells that are responsible for bone resorption and are derived from mononuclear phagocytic cells. Mononuclear precursor cells undergo fusion, thus forming multinucleated osteoclasts during the differentiation process [102]. Differentiation is influenced by various systemic and local factors, including hormones, growth factors, and cytokines, among which the main regulatory pathway for osteoclastogenesis is the RANKL/RANK signalling pathway. When RANKL binds to its receptor RANK on osteoclast precursors, it triggers intracellular signalling pathways, such as the NF-κB pathway [103]. Osteoclasts express an enzyme known as NADPH oxidase, which is responsible for generating ROS. ROS plays a significant role in bone metabolism and is subject to ongoing research in this field. The interaction between RANKL and its receptor RANK triggers the production of ROS in osteoclast cells [104]. The potential involvement of ROS in osteoclast differentiation and bone resorption has prompted the investigation of antioxidants as potential protective agents against bone loss [105]. Oxidative stress has been found to be positively correlated with bone loss, and decreased levels of antioxidants have been observed in patients with osteoporosis. Recent studies have explored the use of metal nanoparticles as potential antioxidants. Metal nanoparticles, such as bimetallic gold and platinum nanoparticles, exhibit antioxidant properties and can scavenge ROS, thus offering potential as protective agents against bone loss. Bimetallic nanoparticles composed of gold and platinum have shown effectiveness in scavenging ROS, including hydrogen peroxide (H_2_O_2_) and the superoxide anion radical (O2-), in a dose-dependent manner [106, 107].

Gold nanoparticles have been shown to possess inhibitory effects on the formation of osteoclasts derived from BMMs when induced by receptor activators of RANKL [73, 103]. Additionally, gold nanoparticles have been observed to reduce the production of ROS and other oxygen radicals induced by RANKL [103, 108]. Additionally, other studies have explored the use of AuNPs conjugated with β-cyclodextrin, which can form inclusion complexes with curcumin. These complexes have demonstrated the ability to suppress osteoclast activation and reduce the occurrence of osteoporosis [101]. In a separate study, the effects of AuNPs on osteoclastogenesis were investigated, thus demonstrating their potential in modulating this process [73]. The researchers observed that AuNPs suppressed preosteoclast fusion induced by receptor activators of RANKL and M-CSF. Moreover, AuNPs significantly inhibited crucial steps in osteoclastogenesis, including cell migration and actin ring formation; in addition, they reduced the bone absorption function of osteoclasts. Notably, the researchers also detected altered expression of fusogenic genes in the treated preosteoclasts. Excessive bone resorption by overactive osteoclasts is a contributing factor to conditions such as osteoporosis [109]. Osteoclasts create an acidic environment within resorption pits by transporting protons, thus facilitating the degradation of the bone matrix by protein hydrolases. Gold nanoparticles have shown potential in inhibiting excessive bone resorption and promoting the repair of bone defects by regulating the acid secretion function of osteoclasts [31]. These findings highlight the potential of AuNPs as therapeutic agents for diseases related to osteoclast-mediated bone metabolism.

### Role of gold nanoparticles in bone formation

One effective approach to improve bone tissue engineering is to promote the osteogenic differentiation and bone-forming capabilities of osteoprogenitor cells. The enhancement of the osteogenic differentiation and bone-forming capabilities of osteoprogenitor cells is a crucial strategy in advancing bone tissue engineering. Mesenchymal stem cells (MSCs) have been identified as being progenitor cells for skeletal tissues, as they possess the ability to differentiate into various lineages, including osteoblasts, chondrocytes, and adipocytes [110, 111]. Nanomaterials can be engineered with specific surface properties, such as topography and chemical composition, which influence cell behavior and promote osteogenic differentiation. Moreover, nanoscale materials can serve as carriers for growth factors and other bioactive molecules, thus facilitating their controlled release and targeted delivery to MSCs. In recent years, a growing body of evidence has indicated the potential of nanoscale materials in facilitating stem cell therapy and bone tissue engineering [112, 113].

**Fig. 3:**
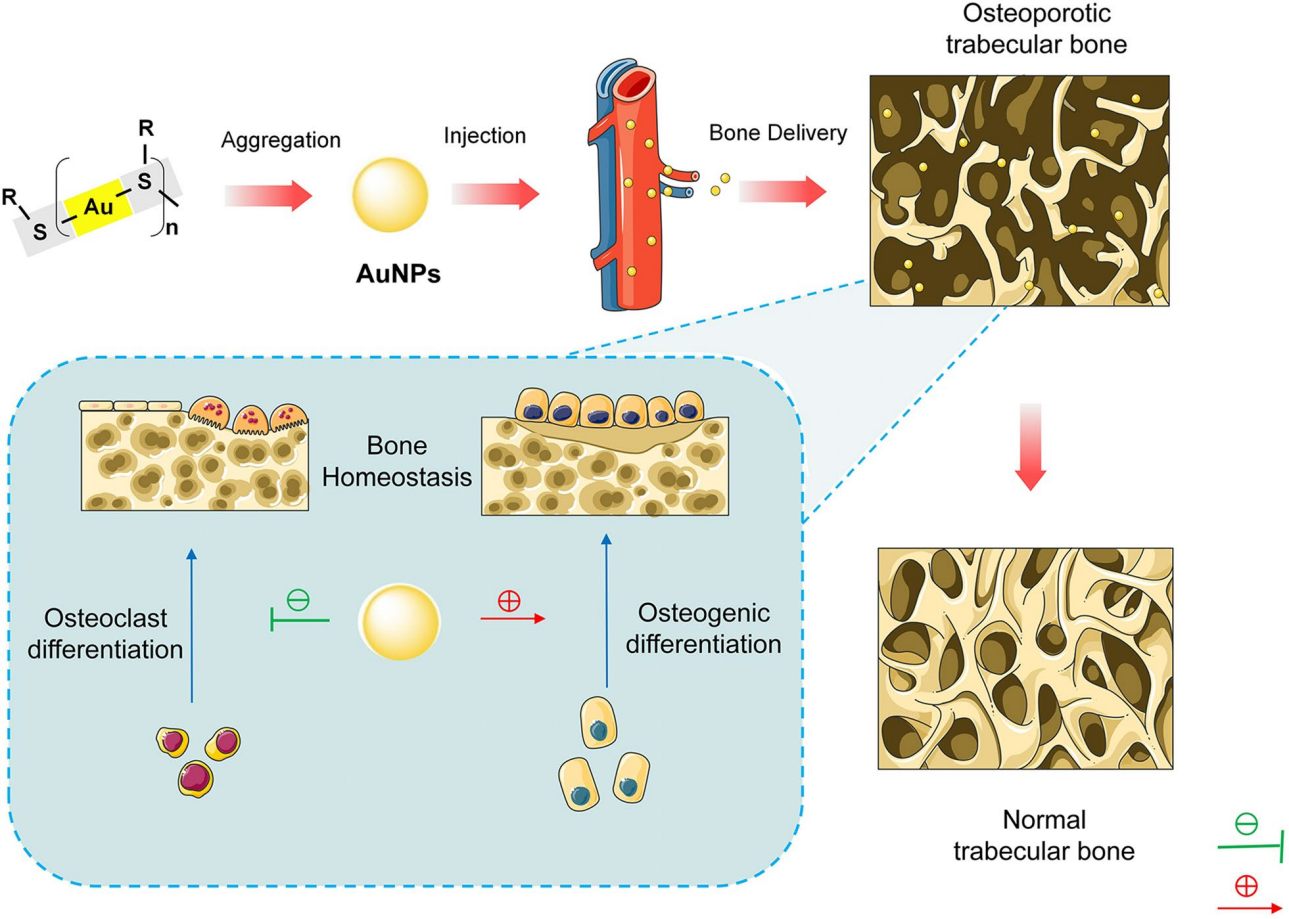
Schematic diagram of the influence of AuNPs on osteogenic and osteoclastic differentiation in bone

Recent studies have demonstrated that gold nanomaterials have a positive impact on bone tissue engineering, which can promote the osteogenic differentiation of BMSCs into osteoblasts, thus enhancing cellular osteogenesis [32, 33]. Mechanical stimuli have also been found to play a crucial role in directing stem cell differentiation, with several signalling molecules implicated in mechanical stress-initiated signal transduction [114, 115]. Among these signalling molecules, MAPK has been shown to be activated by mechanical stresses. The activation of MAPK can link mechanical stress to biochemical responses and gene expression [116]. Moreover, MAPK pathways play critical roles in guiding MSC commitment to the osteogenic lineage [117]. Previous studies have indicated that gold nanoparticles interact with the membrane of mesenchymal stem cells and bind with proteins in the cytoplasm after being internalized through endocytosis, thus exerting mechanical stress on the cells. This mechanical stimulation can correspondingly activate the MAPK signalling pathway, thus leading to the expression of osteogenic genes and the phenotypic differentiation of osteocytes [100, 101]. The Wnt/β-catenin signalling pathway has also been identified as being a key player in osteoblast differentiation [118, 119]. The activation of this pathway inhibits adipogenic differentiation and promotes a shift in cell fate from adipocytes to osteoblasts in human adipose-derived mesenchymal stem cells (hADMSCs) [120]. Gold nanoparticles have been found to promote bone formation by modulating the Wnt/β-catenin signalling pathway [120]. Specifically, AuNPs have been shown to enhance the deposition of calcium content and increase the expression of marker genes associated with osteogenic differentiation in MSCs. This suggests that AuNPs can promote osteogenesis by regulating mRNA expression and mineralization through the Wnt/β-catenin signalling pathway (Fig. 3). AuNPs also possess considerable promise in the field of regenerative medicine due to their biocompatibility and the ability to easily modify their surfaces with biomolecules such as growth factors, DNA, and peptides. These surface modifications and ligands can alter the physicochemical properties of the AuNPs. The osteogenic potential of gold nanomaterials has been well recognized; additionally, to enhance their pharmacodynamic effects, bisphosphonates have been introduced as targeting ligands for gold materials [121, 122]. Bisphosphonate-modified AuNPs have demonstrated increased inhibition of osteoclastic activity and have the potential to be used as an effective treatment for osteoporosis [123]. Vitamin D is a vital nutrient for maintaining normal bone homeostasis, and the use of thiol-polyethylene glycol vitamin D to bind vitamin D to AuNPs offers an attractive advantage in the field of bone tissue engineering [124]. The resulting material, which is known as VD-AuNPs, has shown efficacy in enhancing the osteogenic differentiation of human adipose-derived stem cells (hADSCs) in vitro. In summary, AuNPs are highly promising materials for regenerative medicine due to their biocompatibility and ease of functionalization. By introducing specific ligands such as bisphosphonates or thiol-polyethylene glycol vitamin D, the osteogenic properties of AuNPs can be enhanced, thus making them valuable tools in the field of bone tissue engineering and providing potential solutions for the treatment of bone-related disorders.

## Conclusions and future perspectives

In recent years, nanomedicine has emerged as a promising approach to the treatment of osteoporosis. Various sizes, shapes, and surface-ligand modifications of AuNPs have been developed based on their specific therapeutic effects. For instance, BSA-coated ultrasmall fluorescent gold nanoclusters have been utilized for the treatment of bone loss. In addition, AuNPs can be combined with vesicles [125–127] or incorporated into hydrogel formulations that can be used locally in bone defects with slow-release osteogenic effects [128]. However, it should be noted that AuNPs do not possess inherent targeting abilities. To address this issue, gold composite materials with targeting moieties have been investigated for the treatment of osteoporosis. For example, bisphosphonates and bone-targeting peptides can actively target bone tissue, and gold can be incorporated into their structure to form targeted gold clusters for the treatment of osteoporosis. These findings highlight the promising role of gold nanomaterials in bone tissue engineering and their potential for therapeutic applications in bone-related disorders. This review mainly summarizes the role of AuNPs in osteoporosis. Nevertheless, there are still several challenges to overcome in the development process of AuNPs, including drug metabolism, safety considerations, in vivo efficacy, biocompatibility and stability, preparation costs, and immunogenicity. Furthermore, it is crucial to conduct more in-depth investigations into the underlying mechanisms of AuNPs' effects on osteoclast and osteogenic processes are essential. Despite these challenges, AuNPs continue to hold promise for the treatment of osteoporosis. Building upon the extensive research and successful applications of AuNPs in bioimaging and disease treatment, it is expected that their clinical use in osteoporosis prevention and treatment will continue to advance, thus addressing the current challenges and improving patient outcomes. Our primary goal is to establish a solid scientific foundation for the clinical development of innovative strategies in osteoporosis treatment. However, we acknowledge that this endeavor will be both hopeful and challenging.

## Data Availability

All data will be available upon motivated request to the corresponding author of the present paper.

## Abbreviations

AuNPs: Gold nanoparticles
AuNCs: Gold nanoclusters
ADSCs: Adipose-derived stem cells
BPs: Bisphosphonates
BMMs: Bone marrow-derived macrophages
BMSCs: Bone marrow mesenchymal stem cells
FDA: Food and Drug Administration
GNRs: Gold nanorods
GNSs: Gold nanostars
hADSCs: Human adipose-derived stem cells
hPDLPs: Human bone marrow-derived mesenchymal stem cells
hMSCs: Human mesenchymal stem cells
hPDLCs: Human periodontal ligament cells
MSCs: Mesenchymal stem cells
M-CSF: Macrophage colony-stimulating factor
OPG: Osteoprotegerin
ROS: Reactive oxygen species
RANKL: Receptor activators of nuclear factor-kB ligand
SPR: Surface plasmon resonance
